# The value of post-operative antibiotic therapy after laparoscopic appendectomy for complicated acute appendicitis: a prospective, randomized, double-blinded, placebo-controlled phase III study (ABAP study)

**DOI:** 10.1186/s13063-020-04411-1

**Published:** 2020-06-01

**Authors:** C. Sabbagh, N. Siembida, H. Dupont, M. Diouf, J. L. Schmit, S. Boddaert, J. M. Regimbeau

**Affiliations:** 1 Department of Digestive Surgery, Amiens University Hospital, Amiens University Medical Center, Avenue Laennec, F-80054 Amiens cedex 01, France; 2 Jules Verne University of Picardie, Amiens, France; 3 SSPC (Simplifications des Soins Patients Chirurgicaux Complexes) Research Unit, University of Picardie Jules Verne, Amiens, France; 4 Intensive Care Unit, Amiens University Medical Center, Amiens, France; 5 Department of Methodology, Biostatistics, Direction of Clinical Research, Amiens University Medical Center, Amiens, France; 6 Department of Infectious Diseases, Amiens University Medical Center, Amiens, France; 7 Department of Pharmacology, Amiens University Medical Center, Amiens, France

**Keywords:** Complicated appendicitis, Antibiotic therapy

## Abstract

**Background:**

Approximately 30% of appendectomies are for complicated acute appendicitis (CAA). With laparoscopy, the main post-operative complication is deep abscesses (12% of cases of CAA, versus 4% for open surgery). A recent cohort study compared short and long courses of postoperative antibiotic therapy in patients with CAA. There was no significant intergroup difference in the post-operative complication rate (12% of organ/space surgical site infection (SSI)). Moreover, antibiotic therapy is increasingly less indicated for other situations (non-complicated appendicitis, post-operative course of cholecystitis, perianal abscess), calling into question whether post-operative antibiotic therapy is required after laparoscopic appendectomy for CAA.

**Methods/design:**

This study is a prospective, multicenter, parallel-group, randomized (1:1), double-blinded, placebo-controlled, phase III non-inferiority study with blind evaluation of the primary efficacy criterion. The primary objective is to evaluate the impact of the absence of post-operative antibiotic therapy on the organ/space surgical site infection (SSI) rate in patients presenting with CAA (other than in cases of generalized peritonitis). Patients in the experimental group will receive at least one dose of preoperative and perioperative antibiotic therapy (2 g ceftriaxone by intravenous injection every 24 h up to the operation) and metronidazole (500 mg by intravenous injection every 8 h up to the operation) and, in the post-operative period, a placebo for ceftriaxone (2 g/24 h in one intravenous injection) and a placebo for metronidazole (1500 mg/24 h in three intravenous injections, for 3 days). In the control group, patients will receive at least one dose of preoperative and perioperative antibiotic therapy (2 g ceftriaxone by intravenous injection every 24 h up to the operation) and metronidazole (500 mg by intravenous injection every 8 h up to the operation) and, in the post-operative period, antibiotic therapy (ceftriaxone 2 g/24 h and metronidazole 1500 mg/24 h for 3 days). In the event of allergy to ceftriaxone, it will be replaced by levofloxacin (500 mg/24 h in one intravenous injection, for 3 days). The expected organ space SSI rate is 12% in the population of patients with CAA operated on by laparoscopy. With a non-inferiority margin of 5%, a two-sided alpha risk of 5%, a beta risk of 20%, and a loss-to-follow-up rate of 10%, the calculated sample size is 1476 included patients, i.e., 738 per group. Due to three interim analyses at 10%, 25%, and 50% of the planned sample size, the total sample size increases to 1494 patients (747 per arm).

**Trial registration:**

Ethical authorization by the *Comité de Protection des Personnes* and the *Agence Nationale de Sécurité du Médicament*: ID-RCB 2017-00334-59. Registered on ClinicalTrials.gov (NCT03688295) on 28 September 2018.

## Background

### Appendicitis

The lifetime risk for acute appendicitis (AA) is 7 to 8% [1]. Approximately 30% of these cases are complicated acute appendicitis (CAA; defined as perforated appendicitis, extraluminal fecaliths, an abscess, or local or generalized peritonitis) [2]. Preoperative diagnosis of appendicitis has shifted from suspected appendicitis to proven appendicitis prior to surgery. Preoperative imaging is now the reference for diagnosis and most patients undergo preoperative imaging (ultrasound or computed tomography (CT) scan) [3]. Although appendicitis is a common disease, little is known about its physiology, and the course of the disease (from uncomplicated to complicated appendicitis) is now being questioned.

Complicated and uncomplicated appendicitis may differ in terms of their pathophysiology Complicated appendicitis occurs more frequently in patients with altered inflammatory responses or changes in the colonic microbiome [4]. The results of a recent national cohort study suggest that complicated and uncomplicated appendicitis follow different epidemiological trends over time; the incidence of complicated appendicitis has increased slowly but steadily, whereas non-perforated appendicitis has followed a U-shaped curve. The researchers concluded that complicated and uncomplicated appendicitis were two different diseases, with non-parallel courses and thus non-parallel management [5–7].

Complicated appendicitis is defined as perforated appendicitis, extraluminal fecaliths, an abscess, or local or generalized peritonitis [8]. The definition of localized peritonitis is controversial in terms of the macroscopic aspect of the liquid (from serosity to pus) and the number of quadrants contaminated by pus. It is therefore easier to first define uncomplicated appendicitis (i.e., catarrhal appendicitis without any liquid in the abdomen) and then define complicated appendicitis as any appendicitis that is not uncomplicated. In a recent report on a randomized, controlled trial in children, St Peter et al. defined complicated appendicitis as appendicitis with a hole in the appendix or with fecaliths; they excluded abscesses and local peritonitis from the definition, because the latter conditions were surprisingly associated with a lower rate of post-operative complications than the former (14% vs 18%) [8]. Nevertheless, the study of St Peter et al. is the only one to have specified the nature of complicated appendicitis. All other studies have referred to “complicated appendicitis” without providing any details. More recently, a group at Saint-Antoine Hospital developed a predictive score for CAA (without defining CAA). It is based on five factors (a body mass index (BMI) < 28 kg/m^2^, a leukocyte count < 15,000/ml, a serum C-reactive protein (CRP) level < 30 mg/l, an appendix diameter of 10 mm or less, and the absence of radiological signs of perforation). The likelihood of CAA (ranging from 0% with a score of 5 to 100% with a score of 0) can therefore be used to preoperatively identify patients with CAA [9]. The highest risk for CAA was associated with a Saint-Antoine score ≤ 3. In the present study, we define cases of CAA as patients with a preoperative Saint-Antoine score ≤ 3 that are perioperatively confirmed by the presence of a perforated appendix, extraluminal fecaliths, abscesses, and/or localized peritonitis (pus in one or two abdominal quadrants), according to a recent study of our group [10]. In this study, we found a concordance between surgeons in 85% of cases for the definition of a CAA and the type of CAA (localized or generalized peritonitis) and two quadrants was the best cut-off between localized and generalized peritonitis by receiver operating characteristic (ROC) curve analysis [10]. We have used this definition (two or less quadrants with pus) to define localized peritonitis.

In conclusion, appendicitis is a frequently occurring disease and the Saint-Antoine score (externally validated by our group [7, 11]) helps to identify patients with CAA and thus select patients for the present study.

### Complicated appendicitis

The treatment of complicated appendicitis always includes appendectomy and aspiration of the pus. Exclusive antibiotic therapy is contraindicated.

One of the antibiotic therapies recommended during the preoperative period by both the Société Française d’Anesthésie Réanimation (SFAR) and the Infectious Diseases Society of America (IDSA) is a combination of ceftriaxone and metronidazole (2 g ceftriaxone by intravenous injection every 24 h up to the operation and 500 mg metronidazole by intravenous injection every 8 h up to the operation), with at least one injection before the operation [12, 13]. The IDSA also recommends performing routine aerobic culture tests during the operation to ensure that the antibiotic therapy can be modified in the event of post-operative complications [14].

We have few data (and none from published trials) on the post-operative period. However, available data suggest that post-operative antibiotics are of no value. A Cochrane meta-analysis published in 2005 suggested that the post-operative infection rate was lower in patients who received antibiotic therapy after surgery for AA (odds ratio (OR) [95%CI] = 0.35 [0.13–0.91]; *p* = 0.03). When only cases of CAA were considered, the difference was no longer significant (OR [95%CI] = 0.54 [0.12–2.43]; *p* = 0.4) [15]. However, all the studies included in the Cochrane meta-analysis are more than 20 years old (i.e., published before 1995) and featured laparotomy appendectomy. Laparoscopy was never mentioned and these studies thus no longer provide valid data to address this question (given that 52% of appendectomies are now performed by laparoscopy) [2].

A recent cohort study compared a short (3-day) course of antibiotics with a long course (at least 5 days) for patients with CAA who underwent a laparoscopic or open appendectomy. There was no significant intergroup difference in the overall post-operative complication rate (OR [95%CI] = 1.7 [0.72–4.01]; *p* = 0.2) or the rate of developing an intra-abdominal abscess (OR [95%CI] = 1.77 [0.68–4.58]; *p* = 0.2) [16]. The same authors published a national prospective observational study comparing a 3-day course of antibiotics instead of 5-day course in 2016. They found no difference in the rate of postoperative complications, especially for the rate of developing a postoperative abscess (OR 0.89; 95% CI 0.34–2.35; *p* = 0.81). In this study, the rate of developing a postoperative abscess was 12% [17]. This raises the question of whether post-operative antibiotic therapy is required after laparoscopic appendectomy for CAA. These data are true for all types of complicated appendicitis, except generalized peritonitis, which constitutes a particular group (classified as a high-risk, community-acquired infection by the SFAR). The use of post-operative antibiotic therapy in generalized peritonitis is based essentially on expert consensus, as there are no specific studies supporting this approach. Nevertheless, a 5-day course of antibiotic treatment is required, making generalized peritonitis a special entity and explaining why we excluded this category of patients from the present study [12].

In conclusion, the value of post-operative antibiotic therapy for complicated appendicitis is open to question (except in cases of generalized peritonitis).

### Choice of evaluation criteria

The main post-operative complication for patients with complicated appendicitis after laparoscopy is an organ/space surgical site infection (SSI). In the literature, the incidence of organ/space SSIs for complicated appendicitis (except for generalized peritonitis), i.e., perforated appendicitis, fecaliths outside the appendix, abscess, and localized peritonitis, ranges from 4 to 19%. Most data come from studies that compared open and laparoscopic appendectomy and that did not give details of the post-operative antibiotic therapy. In 1996, Frazee et al. reported an organ/space SSI rate of 7% for patients who underwent laparoscopy, without providing any details of the antibiotic therapy [18]. In 2004, Ball et al. reported an organ/space SSI rate of 4% for patients receiving post-operative ciprofloxacin–flagyl for 7 days after discharge [19]. In 2007, Pokala et al. reported a rate of 14% but did not provide details of the post-operative antibiotic therapy [20]. In 2010, the national cohort study of Tuggle et al. from the USA reported an organ/space SSI rate of 7% in the subgroup of CAA patients who underwent laparoscopy [21]. In 2014, a cohort study of 1143 patients compared 3 with 5 days of post-operative antibiotic therapy and found an organ/space SSI rate of 7.9% [16]. The patients received preoperative intravenous antibiotic therapy (cefozopran hydrochloride, 1 g every 12 h), which was continued into the post-operative period until the inflammatory response abated (based on clinical and laboratory findings, such as fever, pain, bowel movement, oral intake, white blood cell count, and CRP levels). In 2016, Van Rossem et al. reported an organ/space SSI rate of 12% in a national prospective cohort study on patients with localized peritonitis who underwent laparoscopy [17]. We chose 12% as a reference value for patients on post-operative antibiotics to avoid underestimation of the incidence of organ/space SSIs.

In conclusion, the organ/space (deep) SSI rate is the best criterion for evaluating post-operative complications in patients who undergo laparoscopy.

### Choice of antibiotic therapy

The choice of the antibiotic therapy is based on the SFAR and IDSA guidelines adapted to match the bacteriological epidemiology of peritonitis in France. Among the validated therapies, the choice of that used in the protocol was validated by a professor of infectious disease (JLS) and a professor of critical care medicine (HD), who both also participated in writing the SFAR guidelines [12].

In the literature, first-line empirical therapy for peritonitis with no signs of severity must target *Enterobacteriaceae* and anaerobic bacteria. In France, > 75% of the *Enterobacteriaceae* isolated from adult cases of community-acquired peritonitis are susceptible to amoxicillin/clavulanic acid (AMC) [22]. Most cases (90 to 100%) of AMC-resistant *Enterobacteriaceae* are still susceptible to aminoglycosides and third-generation cephalosporins [22]. In France, the prevalence of extended-spectrum beta-lactamase-producing *Enterobacteriaceae* in samples from community-acquired peritonitis in adults is low, and these organisms do not need to be considered in empirical antibiotic therapy (other than in particular local or regional epidemiological contexts). Fluoroquinolones alone are not recommended as a first-line therapy due to a higher rate of resistance [12, 22].

In conclusion, the chosen post-operative antibiotic therapy is ceftriaxone, 2 g/24 h in one intravenous injection, and metronidazole, 1500 mg/24 h in three intravenous injections for 3 days. In the event of allergy to ceftriaxone, it will be replaced by levofloxacin (500 mg/24 h in one intravenous injection, for 3 days).

The aim of this study is to evaluate the use of post-operative antibiotic therapy in complicated appendicitis, with the exception of general peritonitis.

## Methods/design

We hypothesize that post-operative antibiotic therapy is of no value after appendectomy for complicated appendicitis. We expect to observe non-inferiority of the absence of post-operative antibiotic therapy in terms of the organ/space SSI rate in patients presenting with CAA.

### Trial design

This is a prospective, investigational, comparative, double blinded, placebo-controlled, non-inferiority, phase III clinical trial with two parallel groups (randomized 1:1) and a blind analysis of the primary endpoint.

### Inclusion criteria

The ABAP study will include patients with 1) CAA suspected preoperatively and confirmed perioperatively by the presence of a perforated appendix, extraluminal fecaliths, abscesses, and/or localized peritonitis (pus in one or two abdominal quadrants). The time from the onset of symptoms to presentation is not considered. The method of imaging is not imposed (ultrasound or CT scan). There is no official definition of localized CAA. Thus, a training video, based on a previous publication from our group, will be shown at the screening visit to ensure that all investigators have the good definition of localized CAA. The study will also include patients who 2) undergo a laparoscopic appendectomy, 3) are aged ≥ 18 years, and 4) provide a signed written consent form.

### Exclusion criteria

The specific exclusion criteria are detailed in Table 1.

**Table 1:** Exclusion criteria

Preoperative exclusion criteria	
Related to the diagnosis	1) Crohn’s disease, ulcerative colitis, treatment with an immunosuppressive therapy 2) Patients who received an adaptive dose of levofloxacin 250 mg/24 h instead of 500 mg/24 h, preoperatively or perioperatively (notably for patients with creatinine clearance ≤ 50 ml/min), or who have an allergy to metronidazole or one of the excipients and/or who have a contra-indication for the use of ceftriaxone (hypersensitivity to the active substance, to another cephalosporin, to the excipient of the used drug) or who have a history of severe hypersensitivity (such as anaphylactic shock) or hypersensitivity to another antibiotic of the beta-lactam family (penicillin, monobactam, carbapenems) or who have a contraindication for the use of levofloxacin, hypersensitivity to levofloxacin, another quinolone, or the excipient of one of the used drugs, hypersensitivity to levofloxacin or any other quinolone or any excipient, a history of epilepsy, or a history of tendinitis when injected with fluoroquinolones
Related to the severity of the appendicitis	1) Severe sepsis, septic shock, or generalized peritonitis^a^
Related to the treatment	1) A decision to perform open appendectomy or a conversion to open surgery
Perioperative exclusion criteria	
Related to the severity of the appendicitis	1) Non-complicated forms (catarrhal appendicitis or the absence of extraluminal fecaliths, abscess, or peritonitis) 2) Generalized purulent or stercoral peritonitis (the presence of pus or feces in more than two quadrants of the abdomen)

^a^ Severe sepsis is defined as sepsis plus sepsis-induced organ dysfunction or tissue hypoperfusion. Septic shock is defined as persistent sepsis-induced hypotension, despite adequate fluid resuscitation

The other exclusion criteria are related to the research and include: 1) pregnancy or breastfeeding, 2) patients under guardianship, 3) patients unable to give their informed consent, and 4) patients lacking social security coverage.

### Endpoints of the trial

#### Primary outcome

The primary outcome will be the proportion of patients with organ/space SSIs by postoperative day (POD) 30. Organ/space SSIs are officially defined by the CDC [23] as infections that occur within 30 days of surgery AND appear to be related to the surgery AND affect the organ or the cavity around the surgical site (i.e. any anatomical structure—other than the incision—that is opened or handled during surgery) AND for which at least one of the following signs is observed: pus coming from a drain placed in the organ or cavity, bacteria isolated from a liquid or tissue sample collected aseptically from the organ or cavity, and an abscess or other obvious sign of infection of the organ or cavity found by macroscopic examination during subsequent surgery or in a radiological or histopathological examination. Infection of the organ or cavity is diagnosed by the surgeon (or the physician attending to the patient). In the protocol, the SSI will be confirmed on a CT scan by the presence of an organ/space abscess (defined as an enwalled fluid-filled cavity in which the fluid contains gases) [24], which will be treated with antibiotics (either alone or in combination with radiological or surgical drainage, depending on the clinician’s decision).

The investigators evaluating the primary endpoint will be blinded to the treatment group.

#### Secondary outcomes

The secondary outcomes include three types: patient related, infection related, and surgery related.

The patient-related outcomes will include their quality of life prior to surgery, on discharge, and on POD 30, using the EuroQol 5D and SF36 questionnaires.

The principal infection-related outcome will be the proportion of patients with superficial SSIs, defined as infections that occur within 30 days of the intervention and affect the skin and subcutaneous tissues and for which at least one of the following signs is observed: pus coming from the superficial part of the incision, bacteria isolated from a liquid or tissue sample collected aseptically from the superficial part of the incision, a sign of infection (pain, tenderness, redness, burning, etc.) associated with deliberate opening of the superficial part of the incision by the surgeon (except if the culture is negative). Infection of the superficial part of the incision is diagnosed by the surgeon (or the physician attending to the patient). The definitions are based on that published by the CDC. Additional infection-related outcomes include the post-operative infection rates by POD 30, including SSIs and distant infections, the number of antibiotic-free days between randomization and POD 30, a description of the microbial flora, as found in the antibiogram of the perioperative sample that is collected in all cases, and the balance between antibiotic therapy and microbial resistance. The antibiotic treatment will be considered adequate if no bacteria are found in the perioperative sample or if all of the detected bacteria are sensitive to the administered antibiotic therapy. The antibiotic treatment will be considered inadequate if the perioperative sample is positive for resistant bacteria.

Surgery-related outcomes will include morbidity and mortality according to the Dindo-Clavien classification [25] and the CCI [26]; length of stay (LOS), defined as the number of days of hospitalization between surgery and discharge; and rehospitalization rate, defined as rehospitalization during the study period.

### Treatment administered

After randomization, patients in the treatment group will receive at least one dose of preoperative and perioperative antibiotic therapy (ceftriaxone, 2 g via intravenous injection every 24 h up to the operation, and metronidazole, 500 mg via intravenous injection every 8 h up to the operation). In the event of an allergy to ceftriaxone, it will be replaced by levofloxacin (500 mg/24 h in one intravenous injection up to the operation). The perioperative period will start at the beginning of anesthesia and finish at the end of anesthesia. In the post-operative period, patients will receive a placebo for ceftriaxone, 2 g/24 h in one intravenous injection (or a placebo for levofloxacin 500 mg/24 h in one intravenous injection for 3 days, in case of allergy to ceftriaxone), and a placebo for metronidazole, 1500 mg/24 h in three intravenous injections, for 3 days.

Ceftriaxone is a third-generation cephalosporin with broad-spectrum Gram-negative activity. It has low efficacy against Gram-positive organisms but high efficacy against resistant organisms. Furthermore, it is highly stable in the presence of beta-lactamases (penicillinase and cephalosporinase) secreted by Gram-negative and Gram-positive bacteria. The compound’s bactericidal activity results from inhibiting the synthesis of peptidoglycan (a major structural component of the bacterial cell wall) through binding to one or more penicillin-binding proteins. The bacteria eventually lyse because the activity of cell-wall autolytic enzymes continues while cell-wall assembly is arrested. Ceftriaxone is metabolized by the liver. Metronidazole is an anti-infectious nitroimidazole derivative with the highest activity against strict anaerobes.

After randomization, patients in the control group will receive at least one dose of preoperative and perioperative antibiotic therapy (ceftriaxone 2 g by intravenous injection every 24 h up to the operation) and metronidazole (500 mg by intravenous injection every 8 h up to the operation). In the event of an allergy to ceftriaxone, it will be replaced by levofloxacin (500 mg/24 h in one intravenous injection up to the operation). In the post-operative period, antibiotic therapy (ceftriaxone 2 g/24 h and metronidazole 1500 mg/24 h) will be continued for 3 days. In the event of allergy to ceftriaxone, it will be replaced by levofloxacin (500 mg/24 h in one intravenous injection, for 3 days). The antibiotic therapy in the control group is that recommended by the Société Francaise d’Anesthésie Réanimation (SFAR) and the American IDSA guidelines.

### Data collected and follow-up

The screening visit is conducted by the investigating physician. It will take place on the day of the inclusion visit. Before the initiation of any study-related examinations, the investigator will obtain the participant’s written, informed consent. To assess generalizability, all patients fulfilling the eligibility criteria in each center will be recorded in a table.

The screening visit will include a physical examination, including cardiac blood flow, body temperature, and pain evaluation, and a biological examination, including a blood ion profile, and ASAT, ALAT, total bilirubin, indirect bilirubin, alkaline phosphatase, GGT, amylase, lipase, CRP, hCG (if applicable), procalcitonin levels, and leukocyte count.

#### Obtaining consent

At the screening visit, the investigating physician informs the patient about the study and answers any questions the patient may have regarding the study’s objective, nature, constraints, anticipated risks, expected benefits, the placebo, and blinding. The physician also explains the patient’s rights in the context of interventional research involving human subjects and checks the eligibility criteria. A copy of the study information sheet and the consent form are then given to the participant by the investigating physician.

After this information session, the participant has a period of time to consider his/her decision. The investigating physician is responsible for obtaining written, informed consent from the participant. The consent form must be signed before randomization.

If the patient agrees to participate in the study, he/she and the investigator write their names and forenames clearly on the consent form and then date and sign it. Copies of the study information sheet and the consent form are then given to the participant and the original kept by the investigating physician in a closed room, as recommended by national legislation. At the end of the inclusion period (or at the end of the study at the latest), a copy of each consent form is sent to the sponsor or his/her representative via the procedure to be communicated to the investigators in due course.

#### Inclusion visit

The inclusion visit (which is the same as the screening visit) will include a physical examination, including cardiac blood flow, body temperature, and pain evaluation; a laboratory test battery (blood ion profile, ASAT, ALAT, total bilirubin, indirect bilirubin, alkaline phosphatase, GGT, amylase, lipase, CRP, hCG (if applicable), procalcitonin levels, and leukocyte count).

The laparoscopic appendectomy will then be performed, regardless of the hour or day, and the randomization performed after the appendectomy.

#### Follow-up visits

During hospitalization, the patient will be examined twice a day by the attending medical staff. A blood test will be performed on day one (a complete blood count, platelet count, C-reactive protein, and procalcitonin levels; Table 2, Spirit figure).

**Table 2:** Follow-up visits (Spirit figure)

	Screening visit D−1 or D0	Inclusion D−1 or D0	Preoperative period D−1 or D0	Perioperative period	Post-operative period D1–D3	Post-operative FU call D8 post-surgery	Post-operative FU visit D15 ± 7 post- surgery	Post-operative FU visit D30 ± 7 post-surgery
Check on eligibility	✓	✓						
Informed consent		✓						
Clinical examination		✓	✓	✓				✓
Biological examination		✓	✓	✓		✓^a^		
ß-hCG test (women of child-bearing age)		✓						
Search for AEs		✓	✓	✓				✓
Surgery (laparoscopic appendectomy)				✓				
Randomization				✓				
Systematic perioperative sample				✓				
Post-operative antibiotics for 3 days					✓			
Daily follow-up visits				✓				
Post-surgery follow-up surgeon						✓	✓	✓

^a^ blood ion profile, ASAT, ALAT, total bilirubin, indirect bilirubin, alkaline phosphatase, gamma GT, amylase, lipase, CRP, beta HCG, procalcitonin and leucocyte count.

*FU* follow-up

**Fig. 1:**
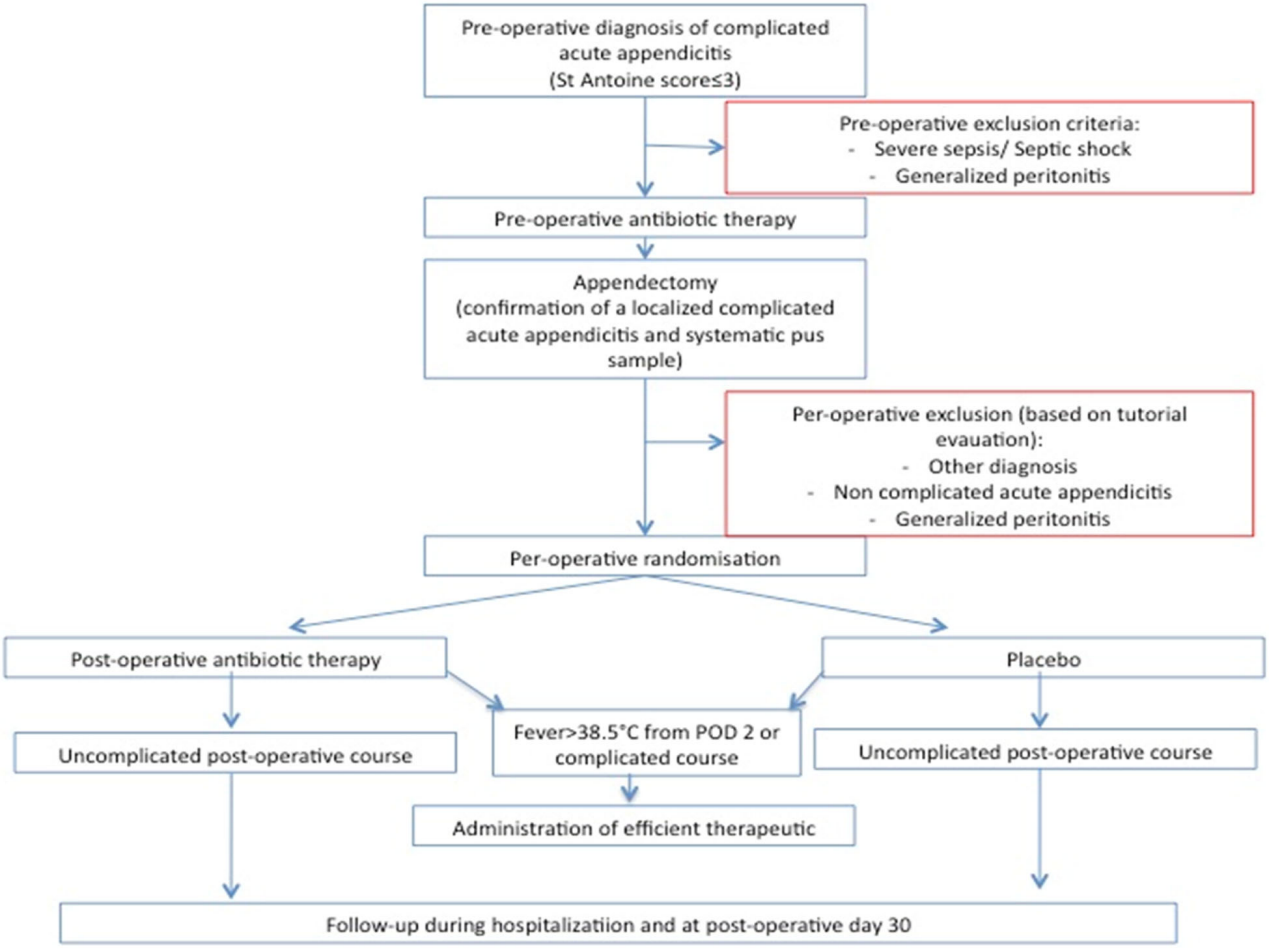
Synopsis of the study

Depending on the clinical situation and the suspected presence of infection, several scenarios are possible. Patients randomized into the antibiotic group can potentially be discharged by the surgeon after completion of the intravenous treatment on POD 4 if there is no post-operative infection. In such an event, the antibiogram will not be inspected by the surgical team. If the patient has a temperature of more than 38.5 °C (suggesting the presence of a post-operative infection), appropriate additional examinations and an antibiogram will be obtained. If a post-operative infection is confirmed and the antibiogram results are available, the antibiotic treatment will be modified. If a post-operative infection is not confirmed, other appropriate treatment will be provided (Fig. 1).

Patients randomized into the non-antibiotic group can potentially be discharged by the surgeon after completion of the intravenous placebo on POD 4 if there is no post-operative infection. If the patient has a temperature of more than 38.5 °C (suggesting the presence of a post-operative infection), appropriate additional examinations and an antibiogram will be obtained. If a post-operative infection is confirmed, an appropriate antibiotic treatment will be administered. If a post-operative infection is not confirmed, other appropriate treatment will be provided.

At the moment of discharge, patients will leave with a letter describing all the signs to monitor that should elicit the patient to seek medical care. He/she will be called by the investigating site on POD 8 to diagnose any sign of complications. Patients will be asked whether they had fever or abdominal pain and be reminded of the phone number of the hospital digestive surgery unit, as given in the discharge letter (available 24 h a day). At the postoperative follow-up visits, the investigator will check for post-operative complications. The first visit will take place 15 days after the patient’s surgery and will include a blood test (a complete blood count, platelet count, C-reactive protein, and procalcitonin levels). The second visit will take place 28 days after the patient’s surgery. During the follow-up visit, the investigator will check on the occurrence of an adverse event and (if applicable) the management of this event (hospitalization, antibiotics, radiological, or surgical drainage, etc.). The primary endpoint will be evaluated at this time.

#### End of follow-up visit

There will be two post-operative visits, on POD 15 and one month (± 1 week) after the patient’s discharge. This visit will be performed by the investigators (and not by the patient’s general practitioner) for all included patients. Patients not seen at POD 15 and/or one month will be invited to visit the hospital. The patient’s general practitioner (who will have received a letter emphasizing the importance of the primary endpoint) will also be contacted before the patient is considered to have been lost to follow-up.

All data will be anonymized.

#### Cessation rules

All patients will be free to withdraw their consent and ask to be withdrawn from the study at any time and for any reason. In the event of premature withdrawal, the investigating physician must specify the reason as fully as possible.

The investigating physician will be able to withdraw a patient for any reason that might harm the patient (especially the presence of an adverse event).

The study may be stopped in the event of unexpected serious adverse events or new information suggesting that the study’s objectives will not be reached, as decided by the sponsor. A data safety monitoring board (DSMB) has been nominated (including a biostatistician, a surgeon, an infectious disease specialist, and an anesthesiologist). There will be a meeting after the first ten inclusions (in case of an adverse event), every 100 inclusions, and at the interim analysis. The DSMB has the power to stop the study.

### Randomization

An interactive web response system will be used to randomize patients using a randomization list with blocks of equal size (with the same number of randomized patients in each group, i.e., antibiotics vs no antibiotics) and stratified by center.

At the end of the surgery, investigators will check the inclusion and non-inclusion criteria. If the patient meets all of the inclusion criteria and none of the non-inclusion criteria, the investigators will log him/her into the electronic case report form and request that a treatment number be allocated to the patient.

The sequence of the study treatments (preoperative and perioperative antibiotic therapy, laparoscopic appendectomy, with or without post-operative antibiotic therapy) will be generated by a data manager using a computer program. The allocation of patients to the different study groups will involve the use of a secure, independent computer to ensure that the investigator cannot interfere with the results of this procedure. Randomization (1:1) will be stratified by center.

Randomization will be performed by an identified investigator. At the end of the surgery, the investigator will log onto the study web site (https://recherche-clinique.chu-amiens.fr/csonline/) using his/her personal username and password. The patient will be registered online and a participant number will be allocated. If the patient satisfies the inclusion/exclusion criteria and stratification criteria, he/she will be randomized, and the investigator will gain access to the treatment number allocated to the patient.

### Blinding method

This will be a double-blind study. In the post-operative period, patients will receive either the antibiotic therapy (ceftriaxone-metronidazole or levofloxacin-metronidazole) or the placebo. Both will be injected intravenously for 3 days. The placebo will be formulated in the same format as the antibiotics. The investigators evaluating the primary endpoint will be blinded to the treatment group, as well as the included patients. The nurses who perform the injection will not be blinded. The vigilance unit must have a copy of the randomization list or a set of envelopes for unblinding.

Unblinding will be performed by the vigilance unit if the occurrence of an unexpected serious adverse effect (SAE) is suspected before declaration to the competent health authorities and annually when annual safety reports are drafted*.*

### Participating centers

The patients will be recruited with help from the *Federation de Recherche EN CHirurgie* (FRENCH) centers that have already participated in another randomized control trial funded by the PHRC (the ABCAL trial, in 2009) [27].

### Statistical methods

#### Sample size

The patients will be randomized into two groups, 1 and 2. Group 1 will not receive post-operative antibiotic therapy (the experimental group) and group 2 will receive post-operative antibiotic therapy (the control group). The groups will be compared in terms of the primary endpoint (i.e., the incidence of organ/space SSIs). The study is based on a non-inferiority analysis.

The hypothesis of the study is that the absence of post-operative antibiotic therapy is at least as good as or similar to the presence of post-operative antibiotic therapy. Furthermore, we assume that the incidence of organ/space SSIs in the reference group is 12%.

The expected organ space SSI rate of 12% in the population of patients with CAA who undergo laparoscopy is based on the publication by Van Rossem et al. [17]. With a non-inferiority margin of 5%, a two-sided alpha risk of 5%, a beta risk of 20%, and a loss-to-follow-up rate of 10%, the calculated sample size is 1476 included patients, i.e., 738 per group. Due to three interim analyses at 10%, 25%, and 50% of the planned sample size, the total sample size increases to 1494 patients (747 per arm). The sample was calculated using the equation on page 290 of the book by S Piantadosi (*Clinical trials: A Methodological Perspective* (2nd edition), John Wiley and Sons, Hoboken, 2005).

#### Statistical methods

Qualitative variables will be presented as numbers (percentages) and compared using a chi-squared test with Yates’ correction or (if applicable) Fisher’s test. Quantitative variables will be presented as the means ± standard deviation or medians (interquartile range) and compared using Student’s *t*-test or a Wilcoxon signed-rank test, as appropriate. Kaplan-Meier curves for the time to event will be built and compared using a log-rank test.

A detailed statistical analysis plan will be defined and validated by the study’s scientific advisory board. Any modifications must be made before unblinding of the database and must be validated by the scientific advisory board.

##### Analysis of the primary endpoint

Non-inferiority will be established if the upper limit of the two-sided 95% confidence interval of the difference in the proportion of infections between the two groups (placebo arm – antibiotic arm) is lower than the non-inferiority margin (5%). An intention-to-treat analysis will be followed by a per-protocol analysis. A multiple imputation analysis will be performed if more than 5% of the data are missing for the primary endpoint.

##### Analysis of secondary endpoints

The patient-related endpoint, consisting of quality of life prior to surgery, on discharge, and on POD 30 after surgery, will be assessed in a mixed model analysis of variance with a Bonferonni-Holm closed testing procedure for post hoc analyses.

In terms of the infection-related endpoints, the proportion of patients with superficial SSIs will be assessed using a chi-squared test or Fisher’s test. The post-operative infection rates by POD 30, including SSIs and remote infections, will also be assessed using a chi-squared test or Fisher’s test. The number of antibiotic-free days between randomization and POD 30 will be assessed using Student’s *t*-test or a Wilcoxon test. The microbial flora will be assessed using a chi-squared test or Fisher’s test. The proportion of patients with adequate antibiotic treatment will also be assessed using a chi-squared test or Fisher’s test.

In terms of the surgery-related endpoints, morbidity and mortality (according to the Dindo-Clavien classification) will be assessed using a Wilcoxon test, LOS will be assessed using a chi-squared test or Fisher’s test, and the rehospitalization rate will be assessed using a chi-squared test or Fisher’s test.

Statistical analysis will be performed using SAS® software (version 9.4, SAS Institute Inc., Cary, NC, USA).

### Ethical considerations

The sponsor and the investigators undertake to ensure that the study is performed according to the French Public Health act 2004–806, dated August 9 2004, Good Clinical Practices (ICH version 4, dated May 1 1996, and the decision dated November 24 2006), and the Declaration of Helsinki (a complete version of which may be found at: http://www.wma.net). Ethical authorization has been given by the *Comité de protection des personnes* and *Agence nationale de sécurité du médicament*: ID-RCB 2017–00334-59.

The study will be conducted according to the present protocol. With the exception of emergency situations requiring the implementation of specific therapeutic procedures, the investigators undertake to comply with every aspect of the protocol, particularly those concerning obtaining the patient’s informed consent and the notification and follow-up of serious adverse events (surgical site infection, pneumonia, fever > 38.5 °C, post-operative hemorrhage, venous thromboembolism, pulmonary embolism, myocardial infarction, or stroke).

In accordance with the provisions of Article L1121–10 of the French Public Health Code, the study sponsor (Amiens University Hospital) has taken out a civil liability insurance policy with *Société Hospitalière d’Assurance Mutuelles* (18 Rue Edouard Rochet, F-69372 Lyon cedex 08, France; policy number 147731 RC RECH. BIOMEDICALES).

Data recorded during this study will undergo automated processing by the Amiens University Hospital Biostatistics Unit, in accordance with the French Data Protection Act 78–17, dated January 6, 1978, and the French Data Protection Act 2004–801, dated August 6, 2004.

This study falls within the scope of the “Reference Methodology” (*Méthodologie de référence*, MR-001) described in article 54, paragraph 5 of the French Data Protection Act 78–17, dated January 6, 1978 (amended). This modification was approved by the decision dated January 5, 2006. Amiens University Hospital has signed to comply with this Reference Methodology.

In accordance with Article L1121.15 of the French Public Health Code, the present study is registered in the European EudraCT database under No. 2017–000334–59. This study was also registered on the ClinicalTrials.gov website (http://clinicaltrials.gov/; NCT03688295) on September 28, 2018.

The biological sample collection performed in the context of this research was registered with the ANSM at the same time as the application for a clinical trial authorization. After termination of the study, storage of the biological sample collection will be registered with the French Ministry of Research and the Regional Health Agency (*Agence Régionale de Santé*). If the purpose of the research changes, approval from the CPP (IEC/IRB) will be requested.

## Discussion

In 2014, the world Health Organization published a report on the escalating global incidence of multidrug resistance caused by antibiotic overuse, which has become a significant threat to public health worldwide [28]. For example, antibiotic use is a risk factor for *Clostridium difficile* infections. In addition, prolonged antibiotic therapy after surgery can cause nausea, allergic reactions, and digestive complaints. Extended intravenous antibiotic prophylaxis leads to the prolongation of hospital stays and increased costs. There has thus recently been a tendency to minimize the use of postoperative antibiotics for uncomplicated appendicitis and anorectal abscesses [28, 29].

In this field, complicated appendicitis is a frequently occurring disease and accounts for 30% of all appendicitis cases. Thus, there is a real need to address this question. In this context, the first point to resolve was the definition of CAA, for which there are few data. St Peter et al. defined complicated appendicitis as appendicitis with a hole in the appendix or with fecaliths. This excluded abscesses and local peritonitis from the definition because the latter conditions were surprisingly associated with a lower rate of post-operative complications than the former (14% vs 18%, respectively) [8].

Another issue to resolve was the definition of localized peritonitis. We thus performed a study to evaluate how surgeons define localized and generalized peritonitis. In this study, we found that the best cut-off to distinguish localized from generalized peritonitis for surgeons was two quadrants. We also found that the overall concordance between surgeons and the initial operative report was 85% [10].

We also questioned the methodology and use of a placebo in the control group. This was requested by the national fund to limit the bias of the study. However, this increased the complexity of the protocol, as antibiotics and placebo must be prepared at the pharmacy for each patient and blinded from the patient and the surgeons. We first established that nurses should also be blinded. However, it was not feasible, as nurses are reluctant to inject medications prepared by another nurse.

In conclusion, the interest of post-operative antibiotic therapy in CAA is unknown. Therefore, the present trial is designed to evaluate whether a strategy with no post-operative antibiotics is not inferior to a strategy with post-operative antibiotic therapy on organ/space SSIs by POD 30.

## Data Availability

Data sharing is not applicable to this article as no datasets were generated or analyzed during the current study.
